# Ion Channel Disturbances in Migraine Headache: Exploring the Potential Role of the Kynurenine System in the Context of the Trigeminovascular System

**DOI:** 10.3390/ijms242316574

**Published:** 2023-11-21

**Authors:** Eleonóra Spekker, Gábor Nagy-Grócz, László Vécsei

**Affiliations:** 1 Pharmacoidea Ltd., H-6726 Szeged, Hungary; spekker.eleonora@gmail.com; 2 Department of Neurology, Faculty of Medicine, Albert Szent-Györgyi Clinical Center, University of Szeged, H-6725 Szeged, Hungary; nagy-grocz.gabor@szte.hu; 3 Faculty of Health Sciences and Social Studies, University of Szeged, H-6726 Szeged, Hungary; 4 Preventive Health Sciences Research Group, Incubation Competence Centre of the Centre of Excellence for Interdisciplinary Research, Development and Innovation of the University of Szeged, H-6725 Szeged, Hungary; 5 HUN-REN-SZTE Neuroscience Research Group, University of Szeged, H-6725 Szeged, Hungary

**Keywords:** migraine, ion channels, potassium channels, ASICs, purinerg system, kynurenic system, glutamate, trigeminovascular system

## Abstract

Migraine is a primary headache disorder, which is an enormous burden to the healthcare system. While some aspects of the pathomechanism of migraines remain unknown, the most accepted theory is that activation and sensitization of the trigeminovascular system are essential during migraine attacks. In recent decades, it has been suggested that ion channels may be important participants in the pathogenesis of migraine. Numerous ion channels are expressed in the peripheral and central nervous systems, including the trigeminovascular system, affecting neuron excitability, synaptic energy homeostasis, inflammatory signaling, and pain sensation. Dysfunction of ion channels could result in neuronal excitability and peripheral or central sensitization. This narrative review covers the current understanding of the biological mechanisms leading to activation and sensitization of the trigeminovascular pain pathway, with a focus on recent findings on ion channel activation and modulation. Furthermore, we focus on the kynurenine pathway since this system contains kynurenic acid, which is an endogenous glutamate receptor antagonist substance, and it has a role in migraine pathophysiology.

## 1. Introduction

Migraine is a primary headache disorder affecting more than 15% of the world’s adult population during their most productive years, resulting in a global health and economic burden of billions of dollars. The clinical manifestation of migraine involves recurrent attacks accompanied by various associated symptoms [1] Despite intensive research efforts, the underlying processes of the disease are still the subject of ongoing investigations.

Altered ion channel function is implicated in several neurological disorders, and as such, the importance of ion channels in the pathogenesis of migraine has received significant attention in recent decades. Ion channels, especially potassium, sodium, and calcium channels expressed in various regions of the brain, play a role in neuronal signal transmission and the regulation of vascular tone. Dysregulation of these channels may contribute to the processes that trigger migraine attacks. For instance, disruptions in potassium channels can contribute to heightened neuronal excitability [2]. The sudden and synchronized activity of nerve cells, induced by abnormalities in potassium channels, has the potential to lead to headaches and other migraine symptoms [3]. Sodium channels participate in the formation of action potentials [4], while calcium channels regulate the release of neurotransmitters [5]; thus, issues in the regulation of these channels may increase neuronal activity and vascular changes [6]. The exploration of these intricate mechanisms is a long-standing area of research, and this article aims to contribute to mapping out the complexities associated with migraine [7]. In particular, we delve into a detailed examination of the involvement of ion channels and the consequences of their disturbances, seeking to understand how these channels connect to the broader pathophysiology of migraines. Additionally, we conduct a thorough analysis of the structural and functional relationships of various ion channel types to migraines, comprehensively examining the scale from voltage-gated channels to ligand-gated receptors. Our goal is to provide nuanced insights into the chemical processes underlying migraine attacks. Moreover, understanding the role of ion channels in migraine can aid in identifying new therapeutic targets and advancing migraine treatment.

The role of the kynurenine system in the central nervous system (CNS) is complex, and it has recently been associated with migraines. Thus, we are not only focusing on ion channels but also on the kynurenine system to gain a more comprehensive understanding of the pathophysiology of migraines [8]. The interconnection between the nervous system’s ion channels and the kynurenine system provides an opportunity to identify new therapeutic targets and advance the development of treatments for migraine conditions.

## 2. Migraine Pathogenesis and the Impact of the Ion Channels

Migraine is one of the most common neurological disorders, characterized by a moderate or severe headache felt as a throbbing pain on one side of the head. Nausea is common for many migraine patients, with some experiencing vomiting during these episodes. Individuals undergoing a migraine headache tend to become more sensitive to light, sound, and odors. Additionally, some may encounter dizziness or problems with balance during a migraine attack. Furthermore, intensive exercise and physical exertion can exacerbate the severity of headaches [1]. It affects more than one billion individuals across the world, with a 3:1 prevalence in women [1]. According to the Global Burden of Disease Study 2016, migraine ranks as the second most prevalent cause of disability [9].

Although certain aspects of the pathomechanism of migraine are not yet known, the most accepted theory is that activation and sensitization of the trigeminovascular system (TVS) are essential during migraine attacks [10]. This leads to the liberation of neurotransmitters like calcitonin gene-related peptide (CGRP), substance P (SP), pituitary adenylate cyclase-activating polypeptide (PACAP), and neurokinin A (NKA) from primary sensory neurons. These neurotransmitters trigger mast cell degranulation and plasma extravasation [11,12]. Simultaneously, second-order neurons become activated in the caudal trigeminal nucleus (TNC), and their axons ascend to the thalamus, projecting nociceptive information to the primary somatosensory cortex [13].

Some migraineurs experience an aura during migraine attacks, which is a manifestation of temporary visual and somatosensory disturbances caused by cortical spreading depression (CSD)—a slowly spreading wave of depolarization of neurons and glia in the cortex. The aura can encompass not only visual and sensory symptoms but also motor and brainstem symptoms, such as muscle weakness, speech problems, dizziness, or balance disturbances [14,15]. It has been suggested that high extracellular levels of glutamate and K^+^ may be responsible for the propagation of CSD [16]. CSD can activate sensory neurons in the trigeminal ganglia (TG), suggesting the central (CNS) and peripheral nervous system (PNS) have a role in migraine [17]. Following CSD, molecules such as ATP, glutamate, K^+^, H^+^, arachidonic acid (AA), and nitric oxide (NO) are released locally and are thought to diffuse to and activate meningeal nociceptive neurons [18,19,20]; this leads to a localized rise in neuroactive inflammatory mediators and the sensitization of brainstem regions relevant to pain [21,22].

**Figure 1:**
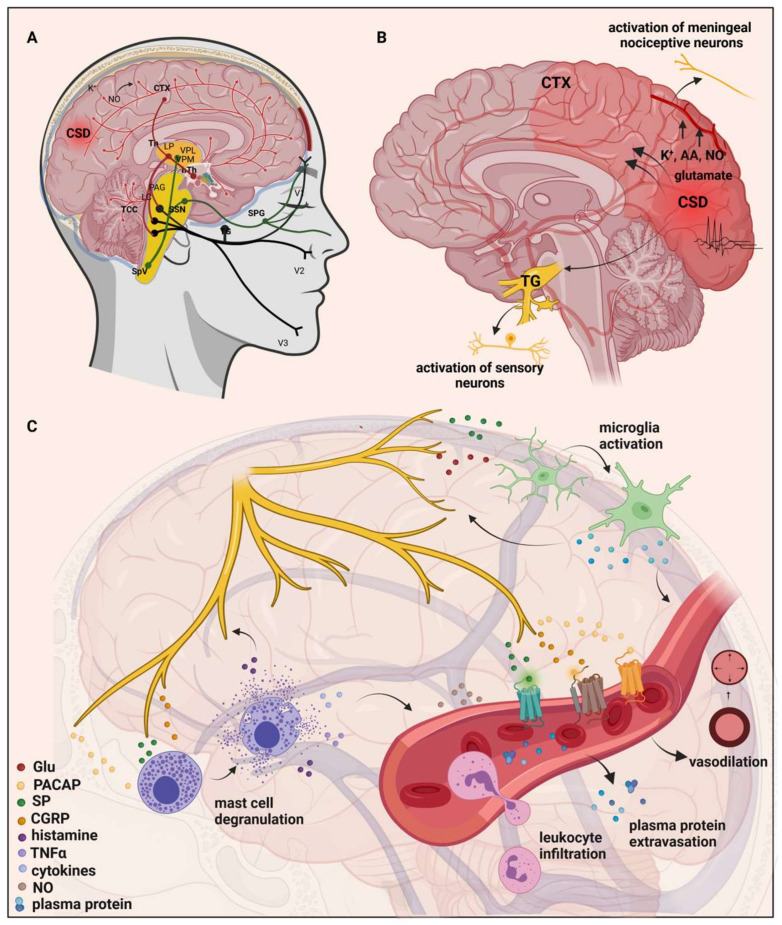
Mechanisms and structures involved in the pathogenesis of migraine. (**A**) Many brain regions are affected during migraine, such as the dorsolateral pons and dorsal midbrain: NRM, DR, LC, and PAG. These nuclei may influence the activity of the TNC and are involved in pain transmission. Moreover, apart from the TVS, they have a two-way connection with the thalamus and hypothalamus. (**B**) Initiation and propagation of CSD are determined by massive increases in extracellular K^+^, NO, and glutamate concentrations. CSD can activate the sensory neurons in trigeminal ganglia, and molecules such as ATP, glutamate, K^+^, H^+^, AA, and NO are released locally and are thought to diffuse to and activate meningeal nociceptive neurons. As a result, there is a local increase in neuroactive inflammatory mediators and sensitization of brainstem regions relevant to pain. (**C**) Stimulation of the trigeminal nerve causes the release of neuropeptides, leading to neurogenic inflammation. It has four main features: vasodilation and increased vascular permeability, leukocyte infiltration, activation of glial cells, and mast cell degranulation which results in increased production of inflammatory mediators such as cytokines and chemokines. AA, arachidonic acid; CTX, cortex; NO, nitric oxide; CSD, cortical spreading depression; Th, thalamus; hTh, hypothalamus; LP, lateral posterior nucleus; VPM, ventral posteromedial nucleus; VPL, ventral posterolateral nucleus; PAG, periaqueductal grey matter; LC, locus coeruleus; TCC, trigeminocervical complex; SSN, superior salivatory nucleus; SpV, spinal trigeminal nucleus caudalis; TG, trigeminal ganglion; SPG, sphenopalatine ganglion; V1, ophthalmic nerve; V2, maxillary nerve; V3, mandibular nerve; Glu, glutamate; CGRP, calcitonin gene-related peptide; SP, substance P; PACAP, pituitary adenylate cyclase-activating polypeptide; TNFα, tumor necrosis factor alpha; NRM, nucleus raphe magnus; DR, nucleus raphe dorsalis.

The trigeminocervical complex (TCC) makes direct connections with the periaqueductal gray (PAG) and areas of the rostral ventromedial medulla (RVM), including the nucleus raphe magnus (NRM), nucleus raphe dorsalis (DR), and locus coeruleus (LC) [12,23]. These nuclei affect TNC activity, and they have a role in pain transmission [24,25]. In addition, the TCC also sends direct projections to higher structures, such as the hypothalamus and thalamus, and from there, the incoming signal projects to the cortex [25]. The hypothalamus establishes direct connections with various structures implicated in pain processing, including the nucleus tractus solitarius, rostral ventromedial medulla, PAG, and NRM [26]. Moreover, dural nociceptive stimulation activates several hypothalamic nuclei [27]. As a result of a dural stimulus, the neurons of the TVS become mechanically hypersensitive; the reason for this may be that the migraine headache is throbbing in nature and intensifies when coughing or bending [12,28] (Figure 1).

In recent decades, the importance of ion channels in the pathogenesis of migraine has received considerable attention, as an altered ion channel function can be observed in many neurological diseases [29]. Dysfunction or abnormal regulation of ion channels can lead to disruption of excitatory–inhibitory balance, neuronal excitability, and peripheral or central sensitization [7]. Genetic studies have identified several ion channel genes, including *CACNA1A*, *ATP1A2*, and *SCN1A*, which encode ion channels and transport proteins, as possible causes or contributors to familial hemiplegic migraine (FHM) [30,31,32,33]. Their function as ion channels and their involvement in ion transport, along with functional experiments in diverse cell and animal models, have played a part in revealing that their malfunction might play a role in cortical hyperexcitability and migraine.

## 3. Ion Channels in Migraine: Unraveling Pathogenesis and Therapeutic Implications

Ion channels are large membrane-spanning proteins that enable the selective transport of ions, such as potassium, calcium, and sodium. They mediate cell excitability and are essential for proper signaling and cell function [7]. Two types of ion channels can be distinguished, which open in response to changes in the membrane potential; these are voltage-gated ion channels (VGICs) and those that are opened by the binding of a ligand, such as a hormone or a neurotransmitter; these are ligand-dependent ion channels (LGICs) [34].

The activity of VGICs is modulated by the membrane potential of the cells. When the channels are open, they allow the movement of ions along an electrochemical gradient across cell membranes [35]. VGICs are selectively permeable to the main physiological ions (Na^+^, K^+^, Ca^2+^, and Cl^−^) and play an essential role in the generation and promotion of information in the form of action potentials in the CNS and PNS, as well as in the cardiovascular system [4].

LGICs mediate fast synaptic transmission in the nervous system and the somatic neuromuscular junction. The binding of a neurotransmitter to an orthosteric site causes a conformational change in the LGICs, and the channels are opened or gated. Gating can be modulated by binding endogenous or exogenous modulators to allosteric sites [36].

**Figure 2:**
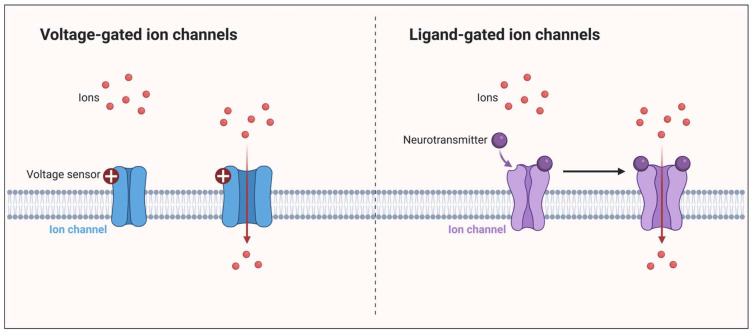
Ion channels: VGICs and LGICs. VGICs have a voltage-sensing domain. After a change in membrane potential, the channel opens and lets the ions flow through. LGICs have a ligand-binding domain. After the binding of the neurotransmitter, a conformational change occurs in the channel, and the free flow of ions occurs through it.

The VGICs allow the permeation of only one type of ion, while the LGICs are less selective and allow the permeation of two or more types of ions through the channel pore [34] (Figure 2).

### 3.1. Potassium Channels

Potassium channels are the largest and most diverse class of VGICs. Potassium channels are located in cell membranes and regulate the flow of K^+^ ions out of and into the cell. The transmembrane protein complexes are involved in the transport of Ca^2+^ ions to mediate or increase the membrane potential.

In the past decade, there has been notable emphasis on the significance of ion channels in the pathogenesis of migraines [37,38,39]. One reason is that ion channels are expressed in cranial arteries and trigeminal afferents and contribute to the regulation of vascular tone and signal transduction in the cephalic pain system [40,41]. Moreover, CGRP and PACAP depend on ion channel activation, particularly potassium channels [42,43]. The discovery of the CGRP and PACAP systems opens up exciting therapeutic possibilities for the future, especially by gaining deeper insights into novel approaches for treating headaches and neurological disorders. This research area has the potential to bring revolutionary changes to healthcare, providing new tools in the fight against such diseases [44,45].

#### 3.1.1. Adenosine Triphosphate-Sensitive Potassium (K_ATP_) Channels

K_ATP_ channels are present both in the PNS and CNS. These channels are widely expressed in the TVS, including the vascular smooth muscle and endothelial cells, the trigeminal ganglion (TG), and TNC, and they play an essential role in regulating the tone of meningeal arteries [37]. These channels inhibit the ATP/ADP ratio at a physiological intracellular level. They activate in response to a decrease in intracellular ATP during metabolic challenges. K_ATP_ channels have a crucial role in the regulation of insulin secretion, vascular tone, and cell protection from metabolic stress [46,47]. There is evidence that K_ATP_ channels are involved in the pathogenesis of migraine.

Among the functions of K_ATP_ channels, the vasodilator effect is particularly important in migraine, as the endogenous neurotransmitters implicated in the onset of migraine attacks are frequently linked to the dilation of cranial arteries [48]. Moreover, several endogenous vasoactive signaling molecules involved in migraine (e.g., CGRP, PACAP, NO, and PGIs) can interact with K_ATP_ channels [37].

CGRP is an endogenous vasodilator molecule present in nerve fibers innervating intracranial vessels [49]. CGRP can indirectly activate vascular smooth muscle K_ATP_ channels through the phosphorylation of adenylate cyclase and protein kinase A (PKA) [50].

Another vasodilator substance is PACAP, which is also found in cerebral arteries [42,49,51]. PACAP can increase intracellular cyclic adenosine monophosphate (cAMP) levels, which activate PKA and induce vasodilation, including through the activation of K_ATP_ channels [42].

Prostaglandin I2 (PGI2) can activate and sensitize meningeal sensory afferents and cause migraine-like attacks in migraineurs. Furthermore, PGI2 enhances K_ATP_ channel activity in vascular smooth muscle through the activation of cAMP-dependent PKA [52].

In addition, the cAMP and cyclic guanosine monophosphate (cGMP) signaling pathways, which play a fundamental role in the development of migraine attacks, are involved in the activation of K_ATP_ channels [53]. The dilation of cerebral and extracerebral arteries through the cGMP pathway is at least partially mediated by the opening of K_ATP_ channels [54]. Based on these, the K_ATP_ channel in the NO-cGMP cascade can lead to a migraine attack.

**Figure 3:**
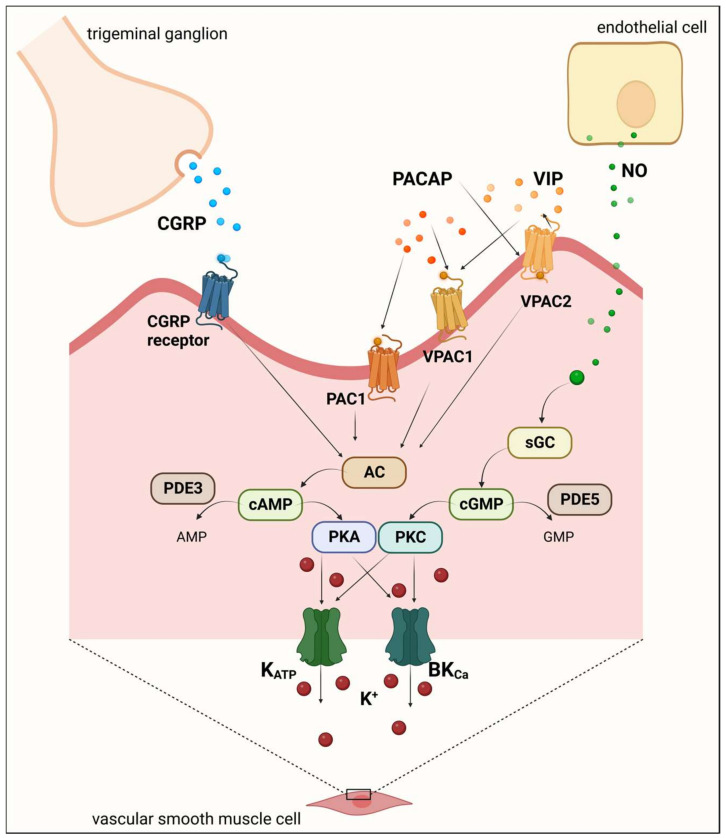
Mechanisms underlying migraine induction: K_ATP_ and BK_Ca_ channel activation. Several endogenous vasoactive signaling molecules involved in migraine (e.g., CGRP, PACAP, and NO) can interact with K_ATP_ and BK_Ca_ channels. These channels are directly regulated by an imbalance between cellular kinase and phosphatase enzymes. PKA and PKG, through the cAMP or cGMP signaling pathways, induce conformation change that activates and opens the channels. The opening of these channels causes a significant efflux of K^+^ and accumulation of extracellular positively charged ions. K_ATP_ and BK_Ca_ channels are involved in the NO/cGMP-dependent signaling pathway and indicate a possible downstream role of these channels in migraine pathophysiology. AC, adenylate cyclase; AMP, adenosine monophosphate; BK_Ca_, large-conductance calcium-activated potassium channel; cAMP, cyclic adenosine monophosphate; cGMP, cyclic guanosine monophosphate; CGRP, calcitonin gene-related peptide; sGC, soluble guanylyl cyclase; K_ATP_, adenosine triphosphate-sensitive potassium channel; NO, nitric oxide; PAC1, pituitary adenylate cyclase-activating polypeptide type 1 receptor; PACAP, pituitary adenylate cyclase-activating polypeptide; PKA, protein kinase A; PKG, protein kinase G; PDE, phosphodiesterase; VIP, vasoactive intestinal polypeptide; VPAC, vasoactive intestinal polypeptide receptor.

As a K_ATP_ channel-opening substance, levcromakalim is the strongest headache and migraine trigger ever studied [53,55,56]. Levcromakalim probably induces migraine by dilating the cranial arteries. Furthermore, levcromakalim induced aura in patients with migraine with aura. The underlying mechanism may be that levcromakalim increases the extracellular K^+^ concentration in neurons, glial cells, and the cerebral vasculature, which causes depolarization in neighboring cells, thus triggering a wave of CSD [57] (Figure 3).

Based on these, K_ATP_ channels may play an important role in the pathogenesis of migraine and may be potential new therapeutic targets in the fight against migraine.

#### 3.1.2. Large-Conductance Calcium-Activated Potassium (BK_Ca_) Channels

BK_Ca_ channels have an essential role in the regulation of neurotransmitter release and vascular tone [58]. These channels manifest their expression in vascular smooth muscle cells found in both extra- and intracranial arteries, as well as in the TG and the TNC [40,59,60].

The BK_Ca_ channel function is controlled by changes in the concentration of intracellular Ca^2+^, membrane potential, and phosphorylation. In addition to these, BK_Ca_ channels are directly regulated by an imbalance between cellular kinase and phosphatase enzymes. PKA and PKG, through the cAMP or cGMP signaling pathways, induce conformation change that activates and opens BK_Ca_ channels [61], so it is conceivable that they have a role in the migraine signaling pathway.

Recently, BK_Ca_ channels were shown to influence neuronal firing in the TNC using a model with dural trigeminovascular nociceptive input [62].

High extracellular K^+^ concentrations have been shown to inhibit NO-mediated vasodilation. Furthermore, NO can directly activate the BK_Ca_ current, even though guanylate cyclase is inhibited [54,63]. Based on these, BK_Ca_ channels may play an important role in the NO/cGMP-dependent signaling pathway and thus in the pathophysiology of migraine.

The infusion of MaxiPost, a BK_Ca_ channel opener, triggers headache in healthy individuals [64]. Other BK_Ca_ channel openers used to treat bronchial asthma, such as andollast and cilostazol, have been associated with headache. It is well known that cilostazol induces headaches in healthy volunteers and migraine-like attacks in migraineurs [64,65]. Another BK_Ca_ opener, iberiotoxin, caused enhanced CGRP release from presynaptic trigeminal fibers in the TNC [60] (Figure 3).

In preclinical studies, several non-selective BK_Ca_ channel-blocking substances, including iberiotoxin, paxillin, and charybdotoxin, were used, which were able to inhibit the physiological effects induced by CGRP and PACAP [3,40,66]. Nevertheless, these blockers lack approval for clinical utilization.

#### 3.1.3. Two-Pore Domain (K_2_P) Potassium Channel

The K_2_P channels represent a varied group of potassium-selective ion channels that play a role in generating background or leak currents in both excitable and non-excitable tissues [67]. Within the human genome, there are 15 genes (KCNK) encoding K_2_P channels. These genes can be categorized into six distinct subfamilies based on both their structural and functional characteristics, specifically the tandem of P domains in a weak inward rectifying K^+^ channel (TWIK), TWIK-related acid-sensitive K^+^ channel (TASK), TWIK-related K^+^ channel (TREK), tandem pore domain halothane-inhibited K^+^ channel (THIK), TWIK-related alkaline pH-activated K^+^ channel (TALK), and TWIK-related spinal cord K^+^ channel (TRESK) subfamilies [67].

The occurrence of members of the TREK subfamily has been thoroughly mapped in both rodents and humans. The expression of TREK-1 and TREK-2 is particularly high in neurons of the CNS during both embryonic and adult stages [68]. In the adult mouse CNS, TREK-1 is primarily found in the cerebral cortex, striatum, hypothalamus, hippocampus, and amygdala [68]. The TREK-2 subunit is predominantly present in the hippocampus, striatum, olfactory bulb, and cerebellar granule cells. Notably, both TREK-1 and TREK-2 are detected not only in neurons but also in cortical astroglial cells [69]. According to a study, TREK-1 and TREK-2 channels are implicated in triggering migraine attacks by regulating TG excitability. Their genetic invalidation induces neural hyperactivity, leading to phenomena similar to migraines, while their activation effectively suppresses migraine-like symptoms induced by NO donors, similar to current migraine drugs targeting neuropeptide release [70]. Therefore, targeting the intrinsic activity of the TREK channels should be considered an alternative strategy for migraine treatment, aiming to reduce TG neuron excitability.

The TRESK channel is widely found in various tissues, especially in neural tissues such as the brain and spinal cord. It is highly expressed in the sensory neurons of the dorsal root ganglion and TG, playing a fundamental role in regulating the excitability of sensory neurons. Its presence in the spinal cord suggests a potential connection to pain pathways [71]. In a TRESK-deficient animal model, increased sensitivity is observed in response to painful mechanical, heat, and chemical tissue-damaging stimuli in the head region. Certain rare mutations of TRESK in humans cause inherited migraines [72]. The role of the frameshift mutation (F139WfsX24) in TRESK in the development of migraine with aura is now well-established [73]. The mechanism has been extensively studied, with some opinions suggesting that the truncated TRESK product originating from an alternative translation initiation site due to the mutation is responsible for inhibiting TREK channels and, in turn, causing migraine [74]. However, others argue that the dominant negative effect exerted on TRESK alone is sufficient for the onset of migraines [72]. Moreover, two mutations (W101R and Y163D + S252L affecting both alleles) have recently been reported, occurring in conjunction with migraine and accompanied by intellectual disability [75]. According to fundamental research results and evidence from animal models, all conditions are present for the activation of TRESK to mitigate the onset or alleviate the symptoms of migraines. There is a need for the development and testing of a selective TRESK activator that is effective even at low concentrations in both animal and human studies. Only based on these results can it be determined with scientific rigor whether activating TRESK could be a therapeutic approach for treating migraines.

### 3.2. Acid-Sensing Ion Channels (ASICs)

ASICs are cation-permeable channels and are activated by increases in the concentration of extracellular protons. Furthermore, it appears that channels can be modulated by both endogenous (neuropeptides, NO, polyamines, and cations) and exogenous (toxins from venoms and amiloride) modulators [76]. The ASIC family consists of four members, ASIC1–4 and six subunits (ASIC1A, ASIC1B, ASIC2A, ASIC2B, ASIC3, and ASIC4). Upon activation, an inward current depolarizes the cell membrane and activates VGSCs, resulting in N-methyl-D-aspartate receptor (NMDAR) activation through the release of the Mg^2+^ blockade [77].

ASICs are expressed throughout the nervous system; their presence has been demonstrated in the spinal cord and several brain regions such as the cortex, hippocampus, periaqueductal grey (PAG), striatum, and amygdala [78,79], suggesting a role for ASICs in the central sensitization of pain.

They are involved in many neurological diseases, including stroke, cerebral ischemia, traumatic brain injury epilepsy, and, based on recent research, also in migraine.

During inflammation, extracellular pH values decrease (below pH = 6), which activates nociceptors by gating ASICs [80].

CSD results in a breakdown of cortical ion homeostasis and the release of H^+^, K^+^, and AA, which are known to potentiate ASICs. Blocking ASIC may inhibit CSD, and thereby aura formation, and prevent subsequent migraine headaches [79].

ASIC3 is highly expressed in sensory neurons and is largely restricted to the periphery [81,82]. ASIC3 is expressed in most trigeminal neurons and is found in approximately 80% of dural afferents [83]. ASIC3 channels are involved in the modulation of various painful conditions, including angina, postoperative pain, various gastrointestinal disorders, and muscle pain [84,85,86]. In relation to migraine, ASIC3 on dural afferents is thought to be a sensor of reduced extracellular pH within the dura [7]. After pH stimulation, CGRP release is also increased in TG neurons via ASIC3 activation, which may result in neurogenic inflammation and migraine progression [87]. The study of Holton and colleagues demonstrates that blocking ASIC3, such as using APETx2, effectively inhibits sensitization of trigeminal nociceptive responses, which is potentiated by the migraine-triggering molecule NO. This discovery supports the development of specific ASIC3 or combined ASIC1/3 blockers for the treatment of migraine-related pain and suggests a potential role in ASIC-dependent NO-mediated migraine triggering [88].

**Figure 4:**
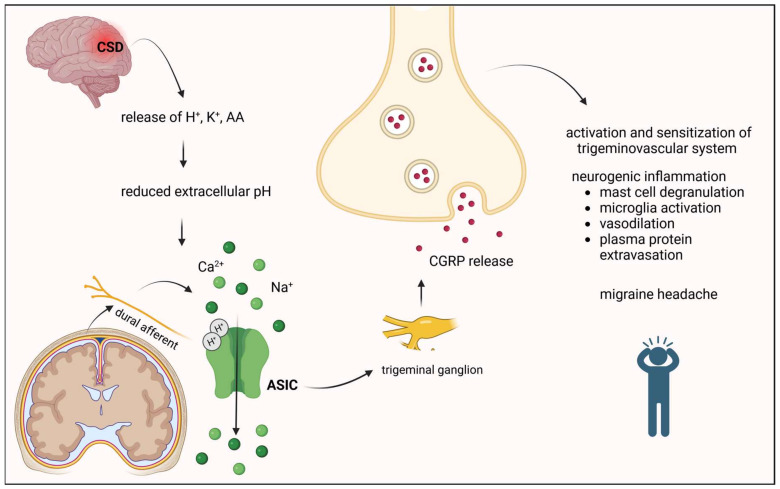
The involvement of ASICs in the process of migraine headache. AA, arachidonic acid; ASIC, acid-sensing ion channels; CGRP, calcitonin gene-related peptide.

In addition to ASIC3, other ASICs may also play a role in the development of migraine attacks. In a preclinical study, amiloride, a nonspecific blocker of ASICs, was shown to block CSD and inhibit trigeminal activation through an ASIC1-dependent mechanism [89]. After peripheral inflammation in spinal dorsal horn neurons, ASIC1 expression increased, and the inhibition of this channel with amiloride reduced pain-related behavioral changes in rodents [90]. Currently, amiloride is undergoing a phase 2 clinical trial to evaluate its effectiveness in the prevention of migraine with aura (Figure 4).

### 3.3. Purinerg System

The purinergic system consists of purinergic receptors, which are divided into two main classes: P1 receptors (adenosine receptors) and P2 receptors (adenosine 5′-triphosphate (ATP) receptors). P1 adenosine receptors are further classified into A_1_, A_2A_, A_2B_, and A_3_ subtypes. Adenosine, a breakdown product of ATP, binds to these receptors and can have inhibitory or excitatory effects on neurotransmission, inflammation, blood vessel diameter, or pain perception [91,92,93,94]. For instance, adenosine’s binding to A1 receptors can inhibit neurotransmitter release [95], while its binding to A2A receptors can have vasodilatory effects [96]. P2 receptors are divided into two main types: P2X receptors (ligand-gated ion channels) and P2Y receptors (G-protein-coupled receptors). ATP, released from various cell types, can activate these receptors, leading to various cellular responses. P2X receptors are responsible for the inflow of cations into the intracellular space of the cell, and they can be found in all mammals. These receptors consist of the heteromeric P2 × 2/3 and P2 × 1/5 receptors, and the homomeric P2 × 1, P2 × 2, P2 × 3, P2 × 4, P2 × 5, P2 × 7 channels.

In the context of migraine, purinergic signaling may influence pain perception, neuroinflammation, and vasodilation, which are all relevant to the pathophysiology of migraine attacks, as described earlier. In pain sensation, P2 × 2, P2 × 3, and P2 × 7 receptors have a distinguished role because they are located in the Aδ- and C-fibers of the primary afferent neurons. This is backed by extensive research. Nociception behaviors in rodents can be provoked by the injection of ATP or αβ-methylene ATP into their skin [97,98], yielding the activation of P2X receptors. In addition to this, P2 × 3 receptor antagonists, namely TNP-ATP (2′,3′-O-(2,4,6-trinitrophenyl)adenosine-5′-triphosphate) and pyridoxal phosphate-6-azo(benzene-2,4-disulfonic acid) (PPADS), can inhibit the acetic acid-induced abdominal constrictions and visceral pain in mice [99]. Besides these findings, an elevated release of CGRP is dependent on activation of the TVS and coexists with a sensitization of P2 × 3 receptors [100]. Furthermore, it has been shown that meningeal purinergic P2 × 7 signaling mediates prolonged meningeal afferent sensitization in a rat model of migraine with aura involving CSD [101]. In fact, our research group demonstrated earlier that the P2 × 7 receptor antagonist Brilliant Blue G attenuates the increase of c-Fos-positive cells in the TNC after the robust electrical stimulation of TG in rats [102].

The role of P2Y receptors in migraine pathomechanism is less known than that of P2X receptors, and the available data show a contradictory picture. The activation of P2Y receptors can cause analgesic and algogenic effects [103], as well. On the one hand, the activation of P2Y1 may block P2 × 3 receptor activity in neurons of the dorsal root ganglia, referring to the anti-algogenic role of ATP and adenosine diphosphate (ADP) [104,105]. On the other hand, the intrathecal administration of uridine-triphosphate (UTP) and uridine-diphosphate (UDP) P2Y receptor agonists has demonstrated analgesic effects, possibly by blocking cytokine release from glial cells [106].

A widely used human and animal model for migraine involves the administration of nitroglycerin (NTG), an agent that releases nitric oxide (NO). NTG activates and sensitizes the trigeminal system [107,108], central mechanism crucial in migraine pathophysiology, as described earlier. In a recent study, it was shown that inhibition of P2Y12 receptors with the selective antagonist PSB-0739 decreases c-Fos expression in the NTG model of migraine pain in mice [109], which underlines the possible role of P2Y receptors in migraine pathomechanism.

**Figure 5:**
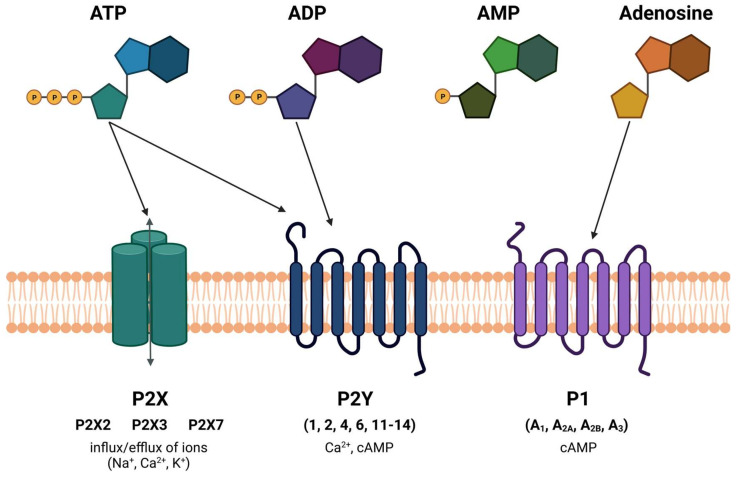
Purinerg system and migraine. P2 × 2, 3, and 7 receptors play a key role in the pathomechanism of migraine. ATP, adenosine 5′-triphosphate; ADP, adenosine diphosphate; AMP, a denosine monophosphate; cAMP, cyclic adenosine monophosphate.

Taken together, P2X and Y receptors may also contribute to the sensitization of the tri-geminal system, and they can modulate the excitability of neurons as well. This increased excitability can result in the perception of pain even in response to mild stimuli, a phenomenon called allodynia. Because of their potential role in migraine pain pathways, P2X receptors can become a target for migraine treatment in the future (Figure 5).

## 4. The Interplay of Glutamate and the Kynurenine Pathway in Migraine

### 4.1. Glutamate and Its Receptors

The glutamatergic system is a crucial neurotransmitter system in the brain that involves the neurotransmitter glutamate. Glutamate is the most abundant excitatory neurotransmitter in the CNS and plays a fundamental role in various brain functions, including learning, memory, cognition, neural plasticity, and pain transmission. The receptors of the glutamatergic system are divided into ionotropic and metabotropic receptors. Ionotropic receptors directly mediate the flow of ions across the cell membrane when glutamate binds to them. The three main types of ionotropic glutamate receptors are NMDA, α-amino-3-hydroxy-5-methyl-4-isoxazolepropionic acid (AMPA), and kainate receptors. Activating these receptors is essential for processes like fast synaptic transmission and synaptic plasticity. Metabotropic receptors are coupled to intracellular signaling pathways through G-proteins and do not directly mediate ion flow. Instead, they modulate neuronal excitability and can have longer-lasting effects on synaptic transmission and plasticity.

Dysregulation of the glutamatergic system has been implicated in various neurological and neuropsychiatric disorders. For example, excessive glutamate release and subsequent overactivation of glutamate receptors can lead to excitotoxicity, a process associated with neurodegenerative diseases like Alzheimer’s disease and Parkinson’s disease, as reviewed by Szalárdy and his colleagues in 2012 [110]. Additionally, abnormalities in the glutamate receptor function have been linked to conditions like schizophrenia, mood disorders, and migraine disorders [111,112,113] as well. Elevated levels of glutamate have been found in the blood and cerebrospinal fluid in patients with migraine [114]. Glutamate excitotoxicity is associated with the hyperexcitability of NMDA receptors [115], which means that high glutamate stimulation causes an excessive amount of calcium ions to enter cells [116]. These processes have a crucial role in damaging DNA and different cell structures, yielding neuronal cell death. These receptors, principally the NMDA receptors, have an essential role in the pathomechanism of migraine.

The exact function of metabotropic receptors of glutamate in relation to migraines is not well understood. However, it is generally accepted that these receptors categorized under group I primarily contribute to the perception of pain [117]. This is because they are situated postsynaptically and, when activated, they heighten the brain’s responsiveness to stimuli. Conversely, metabotropic glutamate receptors in groups II and III are positioned presynaptically, and they work to decrease the release of glutamate, resulting in a mainly pain-relieving effect.

### 4.2. The Kynurenine Pathway

The kynurenine system is a biochemical pathway that involves the metabolism of the amino acid tryptophan. Tryptophan is an essential amino acid, which means that it must be obtained from the diet since the human body cannot synthesize it on its own. The kynurenine pathway is a major route through which tryptophan is metabolized, leading to the production of various metabolites with diverse physiological and immunological functions. The kynurenine pathway starts with the conversion of tryptophan to N-formyl-L-kynurenine by the enzyme indoleamine 2,3-dioxygenase (IDO) or tryptophan 2,3-dioxygenase (TDO), depending on the tissue and the context. N-formyl-L-kynurenine is then further metabolized into L-kynurenine (L-KYN) by formamidase. L-KYN can also be metabolized to kynurenic acid (KYNA) by kynurenine aminotransferases, to anthranilic acid (ANA) by L-kynurenine hydrolase (KYNU), or to 3-hydroxy-L-kynurenine (3-HK) by kynurenine 3-monooxygenase (KMO) as well. ANA and 3-HK are then further degraded to 3-hydroxyanthranilic acid (3-HA), which metabolizes to quinolinic acid (QUIN). 3-HK can be metabolized to xanthurenic acid as well. As the last step of the kynurenine pathway, QUIN is converted to nicotinamide adenine dinucleotide (NAD^+^).

Kynurenines, particularly KYNA, have been identified as endogenous glutamate receptor antagonists. In line with this, KYNA acts as an opposing agent at the strychnine-insensitive glycine-binding site of NMDARs at lower concentrations [118]. Conversely, at higher doses, it also functions by obstructing the glutamate-binding site of NMDA receptors [119]. Furthermore, KYNA elicits mild opposing responses in relation to kainate- and AMPA-sensitive glutamate receptors [117]. Its influence on AMPA receptor-mediated activity is subject to concentration, demonstrating enhancement at lower levels (ranging from nanomolar to micromolar) and inhibition at elevated levels (ranging from micromolar to millimolar) [120]. This Janus-face effect has also been proven by electrophysiological investigations on the hippocampus of young rats, so KYNA actually enhances field excitatory postsynaptic potentials [121].

### 4.3. The Role of Kynurenine Pathway in Migraine Pathomechanism Connected to Glutamate Receptors

Several animal investigations suggest that kynurenines, as well as their analogs and halogenated derivatives, hold promise as potential therapeutic agents for treating migraines. Due to KYNA’s limited ability to traverse the blood–brain barrier, its analogs and derivatives are under experimental evaluation. Specifically, 4,6-dichlorokynurenine and 4-chlorokynurenine halogenated derivatives are converted into KYNA derivatives (7-chlorokynurenic acid and 5,7-dichlorokynurenic acid), which exhibit heightened affinity for the glycine-binding site of NMDA receptors [122,123].

In animal studies, the administration of L-KYN and probenecid (an inhibitor of KYNA secretion from the CNS) or KYNA analogs (N-(2-N,N-dimethylaminoethyl)-4-oxo-1H-quinoline-2-carboxamide hydrochloride (KA1) and N-(2-N-pyrrolidinylethyl)-4-oxo-1H-quinoline-2-carboxamide hydrochloride (KA2) effectively inhibited NTG-induced morphological and behavioral changes, likely by targeting NMDA receptors [124,125,126]. This model revealed decreased expression of kynurenine aminotransferase II (KATII), the primary enzyme in KYNA production, upon NTG administration [127]. Recent research has indicated that NTG influences the expression of other kynurenine pathway enzymes (TDO, IDO, KYNU, and KMO), implying an impact on the kynurenine pathway [128].

Another animal model involving trigeminal activation and sensitization includes the application of Complete Freund’s Adjuvant (CFA) to the dural surface, inducing inflammation. In this setup, KA1 was observed to alleviate CFA-induced inflammation [129]. Moreover, our research group has shown that inflammatory soup could induce sterile neurogenic inflammation in the dura mater, leading to an expansion in the region affected by CGRP and transient receptor potential vanilloid 1 (TRPV1) reactive nerve fibers. Furthermore, there was an increase in the count of neuronal nitric oxide synthase (nNOS)-positive cells in the TNC. Prior applications of KYNA exhibited the capacity to regulate the alterations triggered by the inflammatory soup [130]. In the CFA model, our group also demonstrated that there was a sustained elevation in the levels of glutamate, KYNA, and L-KYN within the TNC 24 h following CFA treatment. Additionally, in the somatosensory cortex, we observed significant increases in the concentrations of KYNA and serotonin, which strengthens the idea that inflammation can influence the elements of the glutamate and kynurenine system [131].

The orofacial formalin test, a model for simulating trigeminal activation and sensitization, demonstrated that probenecid reduced nociceptive behavior in rats by potentially increasing KYNA levels [132]. Recent studies using KA1 and KA2 abolished formalin-induced behavioral and morphological changes, elevating KYNA levels [133]. Additionally, in the combined NTG and formalin model, KA1 inhibited behavioral and morphological alterations [134]. In a trigeminal activation electrical stimulation model, reduced KAT immunoreactivity was observed in the rat’s dura mater [135].

In a CSD model, KA1 and KA2 inhibited CSD wave propagation, likely by targeting glutamate receptors, which play a pivotal role in CSD generation [136], potentially connecting migraine and CSD.

Stimulation of the trigeminal ganglion with electrical impulses led to notable elevations in levels of pituitary adenylate cyclase-activating polypeptide (PACAP)1–38 immunoreactivity, preproPACAP, and PACAP1–38 mRNA within the TNC. These increases were effectively inhibited when rats were pre-treated with KYNA, KA1, and MK-801 [137], which indicates that there is a connection between the kynurenine system and PACAP.

Notably, levels of kynurenine pathway metabolites were found altered in migraine sufferers. Decreased kynurenine metabolite levels were identified in patients with chronic migraine, cluster headache, and episodic migraine [138,139,140,141] consistent with findings from animal studies using the NTG migraine model [127]. These findings suggest that decreased KYNA levels may signify heightened glutamatergic activity in chronic migraine and cluster headache [142].

**Figure 6:**
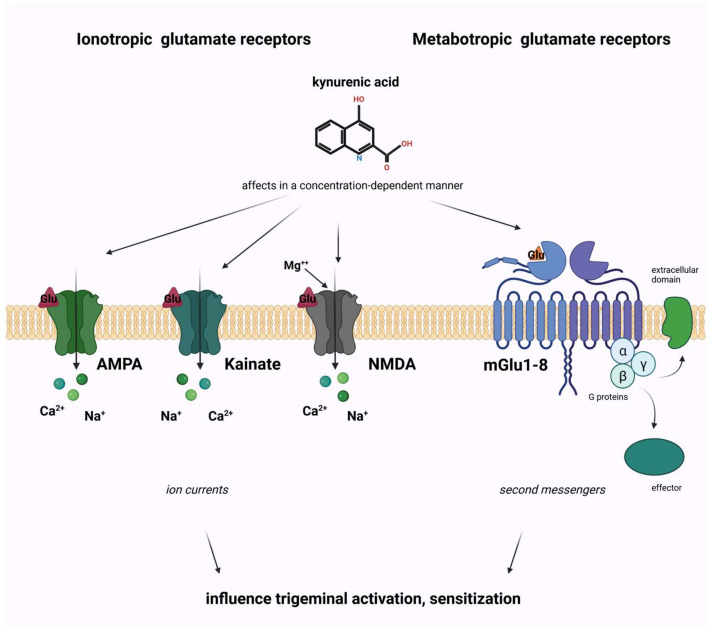
The role of glutamate and kynurenine system in migraine pathomechanism. AMPA, α-amino-3-hydroxy-5-methyl-4-isoxazolepropionic acid; NMDA, N-methyl-D-aspartate; mGlu, metabotropic glutamate receptor.

The precise role of KYNA and its metabolites in migraine pathomechanisms remains partially understood. KYNA’s effects may occur through peripheral and central mechanisms. Peripherally, KYNA can modulate glutamate receptors, particularly NMDA receptors in the dorsal root and TG [143]. Beyond peripheral effects, KYNA and analogs impact second-order neurons, as evidenced by KYNA’s reduction of mechanical allodynia and pain sensitivity in tests like the hot-plate and tail-flick tests [144,145] (Figure 6).

## 5. Conclusions

In summary, these facts indicate that ion channels may play an important role in the pathophysiology of migraine. The activation of primary afferent neurons is prominent in the development of migraine pain, and since several ion channels are expressed on dural afferents, they may contribute to afferent input by sensing environmental changes in the meninges after CSD or inflammatory events. A better understanding of the role of ion channels in migraine attacks may allow the development of new ion channel-based migraine therapies. Moreover, unraveling the intricate connections between ion channels and the kynurenine system may open the door to the development of new and revolutionary migraine therapies. These innovative treatments could prove more effective than currently available options, as they may target the pathophysiology of migraines with greater precision. One less understood aspect of migraine pathology is the mechanism leading to chronification. The mechanism of this transition to chronicity is not yet fully clarified, but numerous factors contribute, including genetic predisposition, excessive use of medications, regular headache attacks, and the presence of other chronic illnesses that can directly or indirectly influence the course of migraine. Thus, the goal is to develop therapeutic strategies that not only reduce migraine attacks but also contribute to preventing or treating the transition to chronic migraine.

## Abbreviations

3-HA	3-hydroxyanthranilic acid
3-HK	3-hydroxy-L-kynurenine
AA	arachidonic acid
ADP	adenosine diphosphate
AMPA	α-amino-3-hydroxy-5-methyl-4-isoxazolepropionic acid
ANA	anthranilic acid
ASICs	acid-sensing ion channels
ATP	adenosine 5-triphosphate
BK_Ca_	large-conductance calcium-activated potassium
cAMP	cyclic adenosine monophosphate
CFA	Complete Freund’s Adjuvant
cGMP	cyclic guanosine monophosphate
CGRP	calcitonin gene-related peptide
CNS	central nervous system
CSD	cortical spreading depression
FHM	familial hemiplegic migraine
IDO	indoleamine 2,3-dioxygenase
KA1	N-(2-N,N-dimethylaminoethyl)-4-oxo-1H-quinoline-2-carboxamide hydrochloride
KA2	N-(2-N-pyrrolidinylethyl)-4-oxo-1H-quinoline-2-carboxamide hydrochloride
KATII	kynurenine aminotransferase II
K_ATP_	ATP-sensitive potassium
KMO	kynurenine 3-monooxygenase
KYNU	L-kynurenine hydrolase
LGICs	ligand-dependent ion channels
L-KYN	L-kynurenine
NAD^+^	nicotinamide adenine dinucleotide
NKA	neurokinin A
NMDA	N-methyl-D-aspartate
NMDAR	N-methyl-D-aspartate receptor
nNOS	neuronal nitric oxide synthase
NO	nitric oxide
NTG	nitroglycerin
PACAP	pituitary adenylate cyclase-activating polypeptide
PAG	periaqueductal grey
PGI2	prostaglandin I2
PKA	protein kinase A
PNS	peripheral nervous system
PPADS	pyridoxal phosphate-6-azo(benzene-2,4-disulfonic acid)
QUIN	quinolinic acid
SP	substance P
TALK	TWIK-related alkaline pH-activated K^+^ channel
TASK	TWIK-related acid-sensitive K^+^ channel
TDO	tryptophan 2,3-dioxygenase
TG	trigeminal ganglion
THIK	tandem pore domain halothane-inhibited K^+^ channel
TNC	caudal trigeminal nucleus
TNP-ATP	2′,3′-O-(2,4,6-trinitrophenol)adenosine-5′-triphosphate
TREK	TWIK-related K^+^ channel
TRESK	TWIK-related spinal cord K^+^ channel
TRPV1	transient receptor potential vanilloid 1
TVS	trigeminovascular system
TWIK	tandem of P domains in a weak inward rectifying K^+^ channel
UDP	uridine-diphosphate
UTP	uridine-triphosphate
VGICs	voltage-gated ion channels
